# Mutual regulation between deubiquitinase CYLD and retroviral oncoprotein Tax

**DOI:** 10.1186/2045-3701-1-27

**Published:** 2011-08-08

**Authors:** Xuefeng Wu, Minying Zhang, Shao-Cong Sun

**Affiliations:** 1 Laboratory of Gene Regulation and Signal Transduction, Department of Pharmacology, School of Medicine, University of California at San Diego, La Jolla, CA 92093, USA; 2 Department of Melanoma Medical Oncology, The University of Texas MD Anderson Cancer Center, 7455 Fannin Street, Box 902, Houston TX 77030, USA; 3 Department of Immunology, The University of Texas MD Anderson Cancer Center, 7455 Fannin Street, Box 902, Houston TX 77030, USA

**Keywords:** CYLD, HTLV, Tax, ubiquitination, IKK, NF-κB

## Abstract

**Background:**

Oncoprotein Tax, encoded by the human T-cell leukemia virus type 1 (HTLV1), persistently induces NF-κB activation, which contributes to HTLV1-mediated T-cell transformation. Recent studies suggest that the signaling function of Tax requires its ubiquitination, although how the Tax ubiquitination is regulated remains unclear.

**Results:**

We show here that the deubiquitinase CYLD physically interacts with Tax and negatively regulates the ubiquitination of this viral protein. This function of CYLD is associated with inhibition of Tax-mediated activation of IKK although not that of Tak1. Interestingly, CYLD undergoes constitutive phosphorylation in HTLV1-transformed T cells, a mechanism known to inactivate the catalytic activity of CYLD. Consistently, a phospho-mimetic CYLD mutant fails to inhibit Tax ubiquitination.

**Conclusion:**

These findings suggest that CYLD negatively regulates the signaling function of Tax through inhibition of Tax ubiquitination. Conversely, induction of CYLD phosphorylation may serve as a mechanism by which HTLV1 overrides the inhibitory function of CYLD, leading to the persistent activation of NF-κB.

## Background

Human T-cell leukemia virus type 1 (HTLV1) is an oncogenic retrovirus that is etiologically associated with a human acute T-cell malignancy termed adult T-cell leukemia (ATL) [1-3]. HTLV1 genome encodes a 40-kD protein that not only regulates viral gene expression but also induces various cellular genes contributing to HTLV1-mediated T-cell transformation [4]. Tax modulates the activity of different cellular transcription factors, most importantly NF-κB, a family of enhancer-binding proteins regulating cell growth and survival [5]. The activity of NF-κB is normally subject to tight regulation by a cytoplasmic inhibitor, IκB. In response to cellular stimuli, IκB is phosphorylated by a specific IκB kinase (IKK) and targeted for ubiquitination and proteasomal degradation, resulting in nuclear translocation of active NF-κB [6,7]. Under normal conditions, the activation of IKK and NF-κB occurs transiently, which assures that the expression of NF-κB target genes is induced temporally. However, in HTLV1-infected T cells, Tax persistently stimulates the activity of IKK, leading to constitutive nuclear expression of NF-κB [8-10]. Strong evidence suggests that deregulated NF-κB activation has a central role in HTLV1-mediated T-cell transformation [5,11,12].

We and others have previously shown that Tax physically interacts with the IKK complex via the IKK regulatory subunit IKKγ (also called NEMO), and this molecular interaction is critical for Tax-mediated IKK activation [13-15]. More recent work suggests that the signaling function of Tax requires its ubiquitination [16-18]. Although ubiquitination is traditionally viewed as a mechanism that mediates protein degradation in the proteasome, it is now clear that specific types of ubiquitination also facilitate the activation of protein kinases, including IKK [19]. In particular, lysine 63 (K63)-linked polyubiquitin chains may serve as a platform that helps recruit and activate IKK and its activating kinase, Tak1. Like phosphorylation, ubiquitination is a reversible reaction, which is counter-regulated by ubiquitinating enzymes and deubiquitinases (DUBs) [20]. A DUB, CYLD, has been shown to preferentially deconjugate K63-linked ubiquitin chains [21] and implicated as a negative regulator of IKK/NF-κB signaling. CYLD has constitutive DUB activity, but its activity can be rapidly inactivated via its phosphorylation in response to NF-κB stimuli [22].

Tax undergos K63 type of ubiquitination, which is critical for activation of NF-κB [23]. However, how the ubiquitination of Tax is regulated remains unclear. In the present study, we have shown that Tax forms a complex with CYLD, in which CYLD strongly inhibits the ubiquitination and signaling function of Tax. Interestingly, in a large panel of HTLV1-transformed T-cell lines, CYLD is constitutively phosphorylated. These findings not only establish CYLD as a negative regulator of Tax ubiquitination but also suggest a mutual regulatory mechanism in which HTLV1 stimulates CYLD phosphorylation and functional inactivation.

## Results

### Tax physically interacts with CYLD

**Figure 1:**
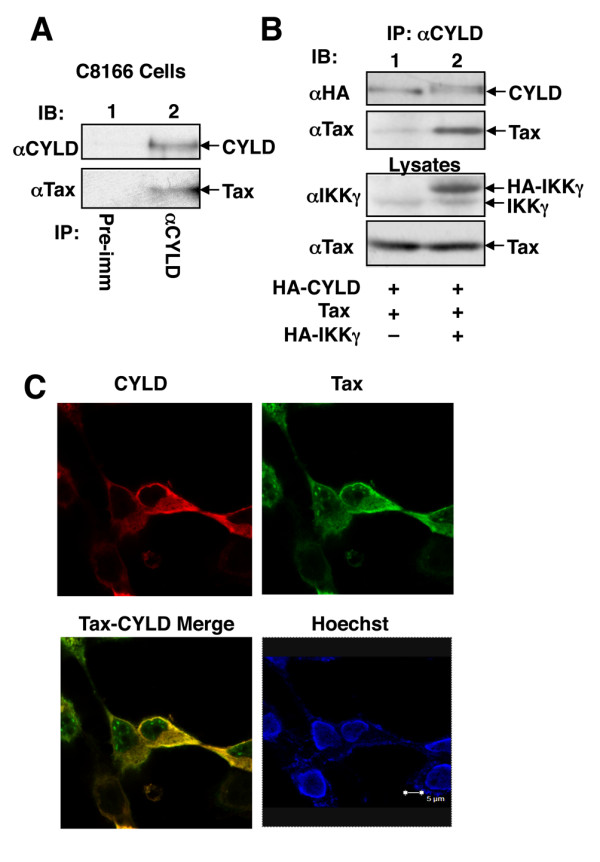
**Physical interaction between Tax and CYLD**. (A) Cell lysates were prepared from the HTLV1-transformed C8166 cell line and subjected to IP using either a control pre-immune serum or anti-CYLD. The precipitated CYLD and its associated Tax were analyzed by IB. (B) HEK293 cells were transfected with HA-CYLD and Tax either in the absence (-) or presence (+) of IKKγ. The cell lysates were subjected to IP using anti-CYLD followed by IB to detect the precipitated CYLD and its associated Tax (top two panels). The cell lysates were also subjected to direct IB to monitor the expression of IKKγ and Tax. (C) HEK293 cells were transfected with HA-CYLD and Tax. The cells were stained with anti-HA (Y-11) and a mouse monoclonal anti-Tax antibody, followed with Texas red-conjugated donkey anti-rabbit Ig and FITC-conjugated donkey anti-mouse Ig. Cells were also counterstained with Hoechst 33258 for nuclear visualization. The expression of CYLD, Tax, Tax-CYLD merge, and nucleus (Hoechst) are shown. Note that the cytoplasmic, but not nuclear, Tax was colocalized with CYLD (Tax-CYLD merge, yellow color).

A prior study suggests that Tax is preferentially conjugated with K63-linked ubiquitin chains [23]. Since CYLD is a K63-specific DUB, we examined whether the ubiquitination of Tax is negatively regulated by CYLD. We first examined the potential physical interaction between Tax and CYLD. In HTLV1-transformed T cells, Tax was readily co-precipitated with CYLD, suggesting that CYLD is present in the Tax complex (Figure 1A). The Tax/CYLD physical association is specific, since a pre-immune serum did not precipitate Tax (Figure 1A). Furthermore, Tax also interacted with CYLD in transiently transfected cells (Figure 1B). Interestingly, the Tax/CYLD interaction appeared to be enhanced by the IKK regulatory subunit, IKKγ, which is known to interact with both Tax [13-15] and CYLD [24,25]. The Tax/CYLD association was also suggested by their colocalization in the cytoplasm of the transfected cells (Figure 1C).

### CYLD inhibits Tax ubiquitination

**Figure 2:**
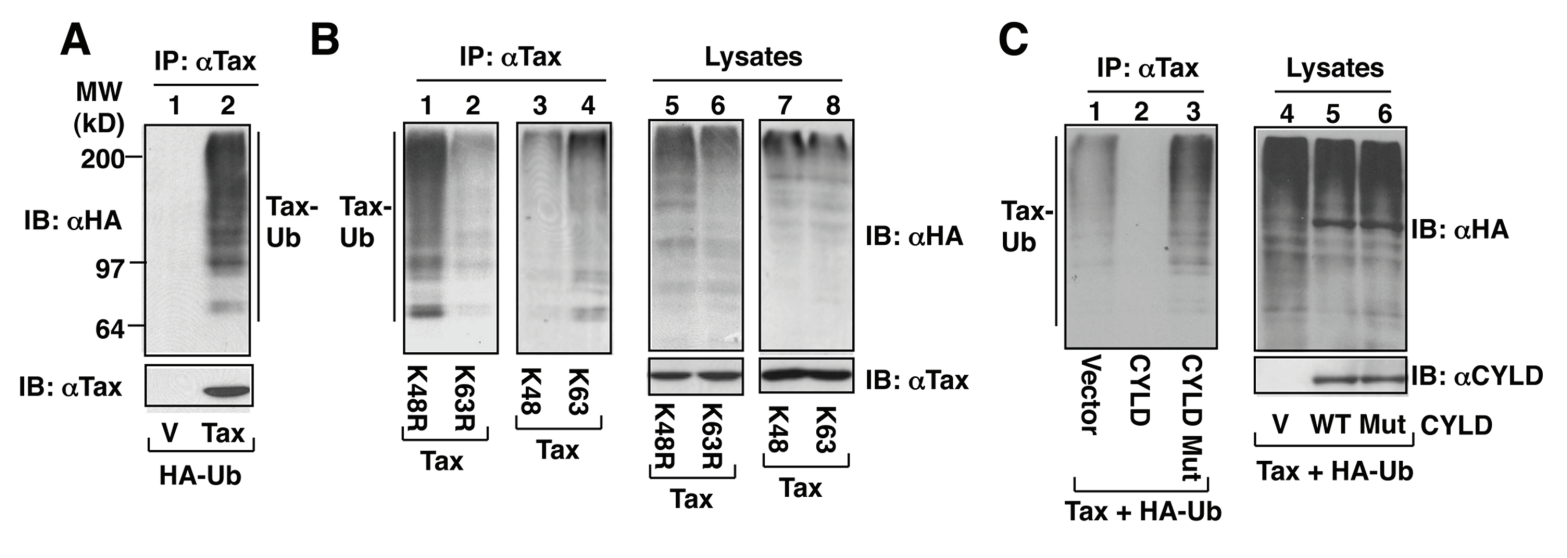
**Tax undergoes K63 ubiquitination, which is inhibited by CYLD**. (A) 293 cells were transfected with HA-tagged ubiquitin together with vector control (V) or Tax. Protein lysates were subjected to IP using anti-Tax, and ubiquitin-conjugated Tax and Tax were detected by IB with HRP-anti-HA and anti-Tax, respectively. (B) 293 cells were transfected with Tax together with the indicated ubiquitin mutants. Tax was subject to IP and ubiquitination assays as in A (left panel). Cell lysates were analyzed by IB to monitor ubiquitin expression (right panels). K48R and K63R harbor K/R substitutions at lysines 48 and 63, respectively. K48 and K63 harbor K/R substitutions in all lysines except 48 and 63, respectively. (C) HEK293 cells were transfected with Tax and HA-tagged ubiquitin either the in the absence (-) or the presence of wildtype (WT) CYLD or a catalytically inactive CYLD mutant (Mut). Cell lysates were subjected to Tax ubiquitination (left panel) and IB (right panel) assays. The Tax expression level was comparable between the different lanes (data not shown).

We next examined whether CYLD regulates the ubiquitination of Tax. As expected, Tax was constitutively ubiquitinated when expressed in 293 cells (Figure 2A). The ubiquitin chains conjugated to Tax appeared to be predominantly K63-linked, since a ubiquitin mutant lacking K63 (K63R) was defective in mediating Tax ubiquitination, whereas the K48R ubiquitin mutant was competent in mediating Tax ubiquitination (Figure 2B, lanes 1 and 2). Furthermore, a ubiquitin mutant retaining lysine 63 but none of the other lysines (K63) was able to mediate Tax ubiquitination (Figure 2B, lane 4). Since CYLD is a DUB that specifically deubiquitinates K63 ubiquitin chains [21,24-26], we tested whether the ubiquitination of Tax is subject to regulation by CYLD. Interestingly, the ubiquitination of Tax was strongly inhibited when it was coexpressed with CYLD (Figure 2C). The inhibitory effect of CYLD on Tax ubiquitination was dependent on its DUB catalytic activity, since a catalytically inactive CYLD mutant failed to inhibit Tax ubiquitination (Figure 2C). These data indicate that CYLD functions as a DUB that negatively regulates Tax ubiquitination.

### CYLD inhibits Tax-stimulated activation of IKK but not that of Tak1

**Figure 3:**
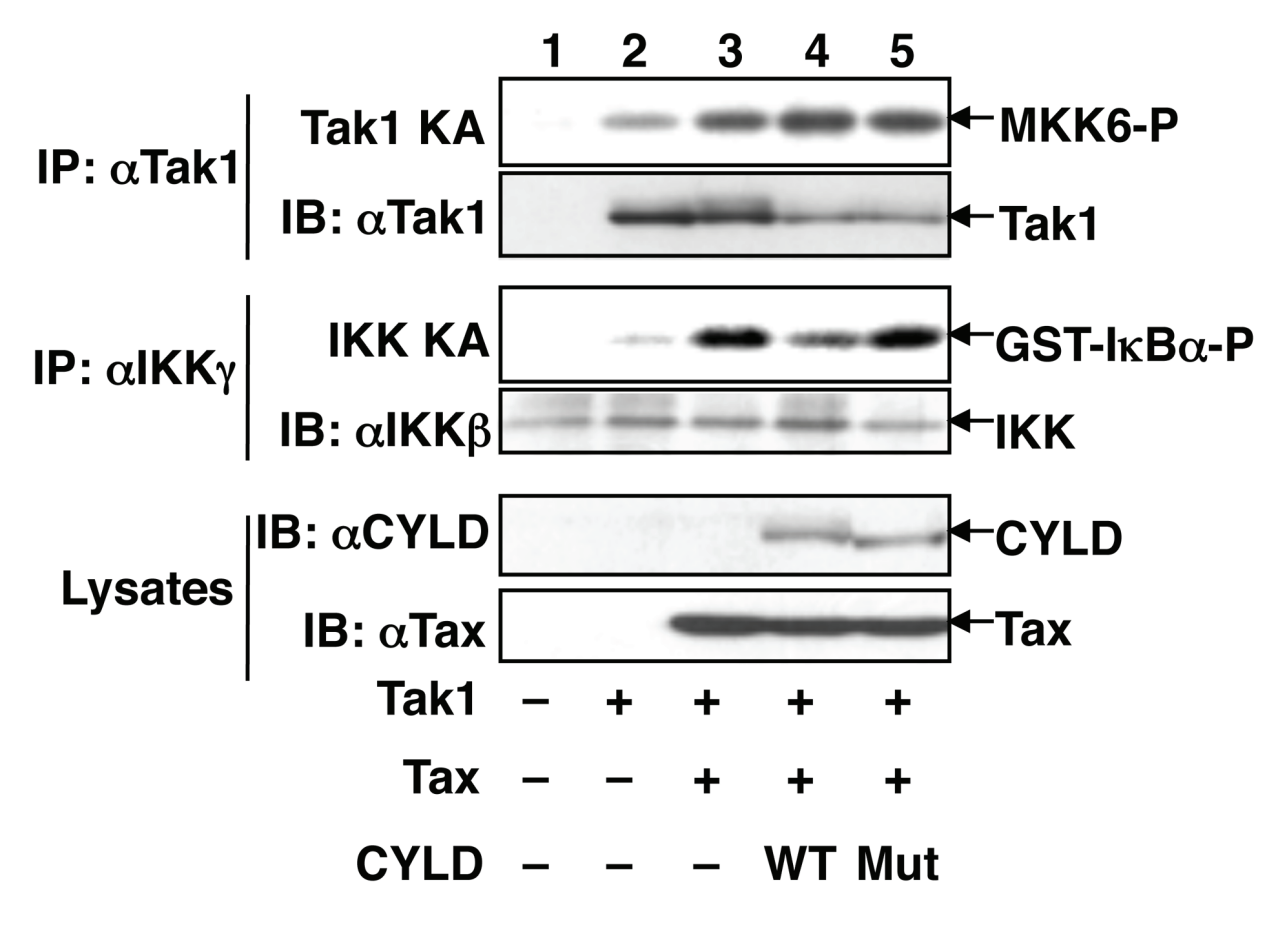
**CYLD inhibits Tax-stimulated activation of IKK but not that of Tak1**. HEK293 cells were transfected with the indicated expression vectors. Cell lysates were subjected to in vitro kinase assays to detect the activation of endogenous Tak1 (panel 1) and IKK (panel 3). Following autoradiography, the kinase assay membranes were subjected to IB to detect the Tak1 (panel 2) and IKKβ (panel 4) proteins. The expression level of CYLD and Tax was monitored by IB (panels 5 and 6).

IKK activation by cellular signals is known to require the MAP3K Tak1 [27-32]. Tak1-mediated phosphorylation of IKKβ, together with a ubiquitin-dependent mechanism, regulate IKK activation by the TCR/CD28 signals [33]. We have previously shown that Tax stimulates the catalytic activity of both Tak1 and IKK [34], although the underlying mechanism remains unclear. Since CYLD deubiquitinates Tax, we examined the effect of CYLD on these Tax-specific signaling events. When expressed in 293 cells, Tak1 displayed a low level of basal catalytic activity as determined by in vitro kinase assay (Figure 3, panel 1). As expected, the kinase activity of Tak1 was potently stimulated by Tax, which was associated with activation of its downstream kinase IKK (Figure 3, panels 1 and 3). Importantly, expression of wildtype CYLD, but not its catalytically inactive mutant, strongly inhibited Tax-stimulated activation of IKK (Figure 3, panel 3), supporting a role for Tax ubiquitination in the activation of IKK signaling. However, to our surprise, CYLD did not affect Tax-stimulated activation of Tak1. Thus, ubiquitination is differentially required for Tax-mediated activation of Tak1 and IKK. This finding is also consistent with our previous observation that Tax-mediated Tak1 activation is required but not sufficient for IKK activation [34].

### CYLD is constitutively phosphorylated in HTLV1-transformed T cells

**Figure 4:**
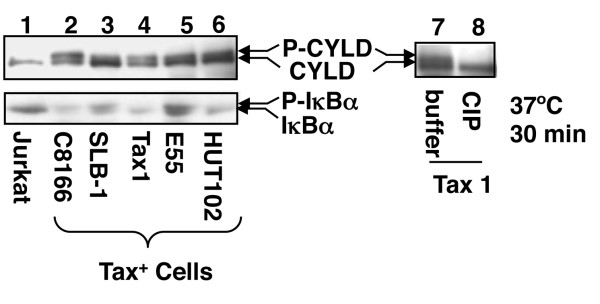
**Constitutive phosphorylation of CYLD in HTLV1-transformed T cells**. Whole-cell lysates were prepared from control Jurkat T cells or the indicated Tax-expressing (Tax^+^) T cell lines transformed by Tax (Tax1) or HTLV1 (C8166, SLB-1, E55, HUT102) and subjected to IB using anti-CYLD (upper) or anti-IκBα (lower). In lanes 7 and 8, the cell lysates were incubated in vitro with either buffer or calf intestinal phosphatase (CIP) to show that the more slowly migrating protein band is phosphorylated CYLD.

The catalytic activity of CYLD is negatively regulated by its phosphorylation [22]. In response to cellular signals, CYLD becomes transiently phosphorylated and inactivated, thus contributing to the activation of IKK. Since Tax stimulates persistent activation of IKK, we examined the status of CYLD phosphorylation in a large panel of T-cell lines transformed by HTLV1 or Tax. As expected, the level of IκBα (a primary target of IKK) was low in these HTLV1-transformed T cell lines (Figure 4). Remarkably, in all of these Tax-expressing cell lines, CYLD was detected as two bands in IB assays (Figure 4). As previously observed in mitogen-stimulated T cells, the upper band represented the phosphorylated CYLD, since it was converted to the basal form upon in vitro phosphatase treatment (Figure 4). Thus, CYLD undergoes constitutive phosphorylation in HTLV1-transformed T cells.

### A phospho-mimetic CYLD mutant failed to inhibit Tax ubiquitination

**Figure 5:**
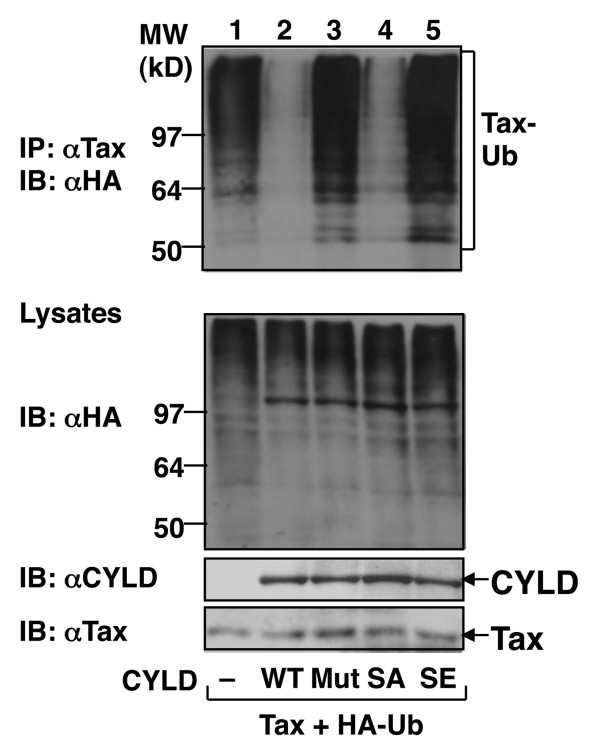
**A phospho-mimetic CYLD is defective in inhibiting Tax ubiquitination**. HEK293 cells were transfected with Tax and HA-ubiquitin either in the absence (-) or presence of wildtype (WT) CYLD, the catalytically inactive CYLD mutant (Mut), or CYLD mutants harboring serine-to-alanine (SA) or serine-to-glutamic acids (SE) substitutions at its phsophorylation sites. Cell lysates were subjected to Tax ubiquitination (upper) or direct IB to analyze the expression of ubiquitin, CYLD, and Tax (lower three panels).

To assess the role of CYLD phoshorylation in regulating its catalytic activity, we examined the effect of a phospho-mimetic CYLD mutant on Tax ubiquitination. As expected, the wildtype CYLD, but not its catalytically inactive mutant, efficiently inhibited Tax ubiquitination (Figure 5, lanes 2 and 3). Importantly, a phospho-mimetic CYLD mutant harboring serine to glutamic acid substitutions at the phosphorylation sites (CYLD7SE, see ref. [22]) completely failed to deubiquitinate Tax (Figure 5, lane 5), whereas a mutant harboring serine to analine mutations at the phsphorylation sites of CYLD (CYLD7SA) remained active in Tax deubiquitination (Figure 5, lane 4). Thus, in HTLV1-transformed T cells, CYLD is inactivated via constitutive phosphorylation, which may contribute to the aberrant activation of IKK and NF-κB.

## Discussion

The data presented in this paper demonstrate a role for CYLD in regulating the ubiquitination of Tax, oncoprotein of the leukemia virus HTLV1 [2]. CYLD is physically assembled into the Tax complex and involved in negative regulation of Tax ubiquitination and signaling function. Furthermore, Tax and CYLD appear to mutually regulate, since CYLD is constitutively phosphorylated in HTLV1-transformed T cells.

Strong evidence suggests that Tax ubiquitination plays a critical role in its signaling function in the NF-κB pathway [35]. Indeed, we have shown that CYLD-mediated Tax deubiquitination is associated with attenuation of IKK activation. Similarly, the ubiquitination and function of Tax are also regulated by another deubiquitinase, USP20 [36]. These findings suggest that ubiquitinated Tax may be targeted by different deubiquitinases, although precisely how the different deubiquitinases regulate Tax during HTLV1 infection remains to be further studied.

Our current study demonstrates that the CYLD-mediated Tax deubiquitination did not affect Tax's ability to activate Tak1. This finding suggests that Tak1 activation is insufficient for Tax-mediated IKK activation, implicating the involvement of different mechanisms in Tax activation of Tak1 and IKK. In further support of this idea, our previous work demonstrates that a Tax mutant, TaxM22, is capable of Tak1 activation despite its defect in IKK activation [34]. One possibility is that Tax not only activates Tak1 but also recruits Tak1 to the IKK complex to mediate IKK activation. Future studies will examine whether Tax ubiquitination is required for recruiting Tak1 to IKK.

The catalytic activity of CYLD is negatively regulated by its phosphorylation [22]. Cellular stimuli induce transient phosphorylation of CYLD, which may contribute to the optimal activation of IKK. A remarkable finding in the present study is that CYLD is constitutively phosphorylated in a large panel of HTLV1-transformed T cells. Since a phospho-mimetic CYLD mutant is defective in inhibiting Tax ubiquitination, the CYLD phosphorylation may contribute to the chronic activation of IKK and NF-κB in HTLV1-transformed T cells. We have previously shown that induction of CYLD phosphorylation by TNF-α or mitogens is mediated by the IKK complex [22]. However, we found that in HEK293 cells, Tax was insufficient for the induction of CYLD phosphorylation (data not shown). This result could be due to the low expression level of IKK components, particularly IKKγ [37], although the possibility for involvement of additional signaling factors cannot be excluded. Regarding the latter possibility, a recent study suggests that CYLD phosphorylation can also be mediated by the IKK-related kinase IKKε and contributes to IKKε-induced tumorigenesis [38]. Whether IKKε is involved in the CYLD phosphorylation in HTLV1-trnasformed T cells is yet to be investigated. It also remains to be examined whether CYLD phosphorylation also contributes to HTLV1-induced T-cell transformation. Nevertheless, our data suggest that CYLD phosphorylation is a mechanism that mediates constitutive Tax ubiquitination and signaling function in HTLV1-transformed T cells.

## Conclusions

The results of this study demonstrate a role for the DUB CYLD in the negative regulation of HTLV1 Tax protein. CYLD inhibits Tax-stimulated IKK activation via deubiquitinating Tax, although the CYLD-mediated Tax deubiquitination does not affect Tax activation of Tak1. Our data further suggest that HTLV1 has developed a mechanism to override the negative-regulatory role of CYLD, and this mechanism involves the induction of CYLD phosphorylation.

## Methods

### Cell lines and transfection

Human embryonic kidney cell line 293, human leukemia T-cell line Jurkat, and HTLV1-transformed human T-cell lines were described previously [34]. The Tax1 cell line is an IL-2-dependent human T-cell line immortalized by HTLV1 Tax in the context of a herpes saimiri vector [39]. For transient transfection, 293 cells were seeded in 6-well plates and transfected using Lipofectamine-2000 (Invitrogen).

### Plasmid constructs, antibodies, and other reagents

pCMV4-Tax was kindly provided by Dr. Warner Greene [40]. pcDNA-based expression vectors encoding HA-tagged IKKγ, Ubiquitin, CYLD, a catalytically inactive CYLD mutant (CYLDmut) were as described [14,22,41]. CYLD SA is a phosphorylation-deficient mutant harboring alanine substitutions of seven serine residues, and CYLD SE is a phospho-mimetic CYLD mutant harboring serine to glutamic acid substitutions [22]. Ubiquitin mutants K48 and K63 (provided by Dr. Zhijian Chen) carry lysine-to-arginine substitutions in all lysine residues, except K48 and K63, respectively. Ubiquitin mutants K48R and K63R harbor mutations in lysine 48 and 63, respectively. pCMV-HA-Tak1 expression vector and anti-Tak1 antibody were kindly provided by Drs. Kunihiro Matsumoto and Jun Ninomiya-Tsuji [42]. All other reagents were described previously [34].

### Immunoblotting (IB), immunoprecipitation (IP), in vitro kinase assay, and ubiquitination assay

Cell lysates were prepared by lysing the cells in RIPA buffer for IB and IP [43]. For kinase assays, cells were lysed in a kinase lysis buffer supplemented with phosphatase inhibitors and immediately subject to in vitro kinase assays as described previously [9]. For ubiquitination assays, cells were transfected with HA-tagged ubiquitin or its mutants along with other expression vectors. The cells were lysed in RIPA buffer supplemented with 4 mM N-ethylmaleimide (NEM) and immediately bioled for 5 min in the presence of 1% SDS and then diluted 10 times with RIPA buffer. Ubiquitinated Tax was isolated by IP using anti-Tax and detected by IB with anti-HA.

### Immunofluorescence assay

HEK293 cells were transiently transfected with HA-CYLD and Tax and subjected to indirect immunofluorescence assays as described [18].

## List of abbreviations

HTLV1: human T-cell leukemia virus type 1; IKK: IκB kinase; DUB: deubiquitinase; IB: immunoblotting; IP: immunoprecipitation; NEM: N-ethylmaleimide

## Competing interests

The authors declare that they have no competing interests.

## Authors' contributions

XW and MZ designed and carried out the experiments and analyzed the data. SCS wrote the manuscript. All authors read and approved the final manuscript.

## References

[B1] Yoshida M Multiple viral strategies of HTLV-1 for dysregulation of cell growth control Annu Rev Immunol 2001 19 475 496 10.1146/annurev.immunol.19.1.475 11244044

[B2] Matsuoka M Human T-cell leukemia virus type I (HTLV-I) infection and the onset of adult T-cell leukemia (ATL) Retrovirology 2005 2 27 10.1186/1742-4690-2-27 15854229 PMC1131926

[B3] Shuh M Beilke M The human T-cell leukemia virus type 1 (HTLV-1): new insights into the clinical aspects and molecular pathogenesis of adult T-cell leukemia/lymphoma (ATLL) and tropical spastic paraparesis/HTLV-associated myelopathy (TSP/HAM) Microsc Res Tech 2005 68 176 196 10.1002/jemt.20231 16276549

[B4] Matsuoka M Jeang KT Human T-cell leukaemia virus type 1 (HTLV-1) infectivity and cellular transformation Nat Rev Cancer 2007 7 270 280 10.1038/nrc2111 17384582

[B5] Sun SC Yamaoka S Activation of NF-κB by HTLV-I and implications for cell transformation Oncogene 2005 24 5952 5964 10.1038/sj.onc.1208969 16155602

[B6] Karin M Ben-Neriah Y Phosphorylation meets ubiquitination: the control of NF-[kappa]B activity Annu Rev Immunol 2000 18 621 663 10.1146/annurev.immunol.18.1.621 10837071

[B7] Hayden MS Ghosh S Shared principles in NF-kappaB signaling Cell 2008 132 344 362 10.1016/j.cell.2008.01.020 18267068

[B8] Chu Z-L DiDonato JA Hawiger J Ballard DW The Tax oncoprotein of human T-cell leukemia virus type 1 associates with and persistently activates IκB kinases containing IKKα and IKKβ J Biol Chem 1998 273 15891 15894 10.1074/jbc.273.26.15891 9632633

[B9] Uhlik M Good L Xiao G Harhaj EW Zandi E Karin M Sun S-C NF-kappaB-inducing kinase and IkappaB kinase participate in human T-cell leukemia virus I Tax-mediated NF-kappaB activation J Biol Chem 1998 273 21132 21136 10.1074/jbc.273.33.21132 9694868

[B10] Yin M-J Christerson LB Yamamoto Y Kwak Y-T Xu S Mercurio F Barbose M Cobb MH Gaynor RB HTLV-I Tax protein binds to MEKK1 to stimulate IkB kinase activity and NF-κB activation Cell 1998 93 875 884 10.1016/S0092-8674(00)81447-6 9630230

[B11] Fu J Qu Z Yan P Ishikawa C Aqeilan RI Rabson AB Xiao G The tumor suppressor gene WWOX links the canonical and noncanonical NF-κB pathways in HTLV-I Tax-mediated tumorigenesis Blood 2011 117 1652 1661 10.1182/blood-2010-08-303073 21115974 PMC3318777

[B12] Qu Z Xiao G Human T-Cell Lymphotropic Virus: A Model of NF-κB-Associated Tumorigenesis Viruses 2011 3 714 749 10.3390/v3060714 21743832 PMC3131208

[B13] Chu Z-L Shin Y-A Yang J-M DiDonato JA Ballard DW IKKγ mediates the interaction of cellular IκB kinases with the Tax transforming protein of human T cell leukemia virus type 1 J Biol Chem 1999 274 15297 15300 10.1074/jbc.274.22.15297 10336413

[B14] Harhaj EW Sun S-C IKKγ serves as a docking subunit of the IκB kinase and mediates interaction of IKK with the human T-cell leukemia virus Tax protein J Biol Chem 1999 274 22911 22914 10.1074/jbc.274.33.22911 10438454

[B15] Jin D-Y Giordano V Kibler KV Nakano H Jeang K-T Role of adaptor function in oncoprotein-mediated activation of NF-κB: HTLV-I Tax interacts directly with IκB kinase g J Biol Chem 1999 274 17402 17405 10.1074/jbc.274.25.17402 10364167

[B16] Lamsoul I Lodewick J Lebrun S Brasseur R Burny A Gaynor RB Bex F Exclusive ubiquitination and sumoylation on overlapping lysine residues mediate NF-kappaB activation by the human T-cell leukemia virus tax oncoprotein Mol Cell Biol 2005 25 10391 10406 10.1128/MCB.25.23.10391-10406.2005 16287853 PMC1291224

[B17] Nasr R Chiari E El-Sabban M Mahieux R Kfoury Y Abdulhay M Yazbeck V Hermine O dT H Pique C Bazarbachi A Tax ubiquitylation and sumoylation control critical cytoplasmic and nuclear steps of NF-kappaB activation Blood 2006 107 4021 4029 10.1182/blood-2005-09-3572 16424386

[B18] Harhaj NS Sun SC Harhaj EW Activation of NF-kappa B by the human T cell leukemia virus type I (HTLV-I) tax oncoprotein is associated with ubiquitin-dependent relocalization of IKK J Biol Chem 2007 282 4185 4192 17145747 10.1074/jbc.M611031200

[B19] Liu S Chen ZJ Expanding role of ubiquitination in NF-κB signaling Cell Res 2011 21 6 21 10.1038/cr.2010.170 PMC3193409 21135871

[B20] Sun SC Deubiquitylation and regulation of the immune response Nat Rev Immunol 2008 8 501 511 10.1038/nri2337 18535581 PMC5763493

[B21] Komander D Lord CJ Scheel H Swift S Hofmann K Ashworth A Barford D The structure of the CYLD USP domain explains its specificity for Lys63-linked polyubiquitin and reveals a B Box module Mol Cell 2008 29 451 464 10.1016/j.molcel.2007.12.018 18313383

[B22] Reiley W Zhang M Wu X Graner E Sun S-C Regulation of the deubiquitinating enzyme CYLD by IkappaB kinase gamma-dependent phosphorylation Mol Cell Biol 2005 25 3886 3895 10.1128/MCB.25.10.3886-3895.2005 15870263 PMC1087725

[B23] Shembade N Harhaj NS Yamamoto M Akira S Harhaj EW The human T-cell leukemia virus type 1 Tax oncoprotein requires the ubiquitin-conjugating enzyme Ubc13 for NF-kappaB activation J Virol 2007 81 13735 13742 10.1128/JVI.01790-07 17942533 PMC2168884

[B24] Kovalenko A Chable-Bessia C Cantarella G Israel A Wallach D Courtois G The tumour suppressor CYLD negatively regulates NF-kappaB signalling by deubiquitination Nature 2003 424 801 805 10.1038/nature01802 12917691

[B25] Trompouki E Hatzivassiliou E Tsichritzis T Farmer H Ashworth A Mosialos G CYLD is a deubiquitinating enzyme that negatively regulates NF-kappaB activation by TNFR family members Nature 2003 424 793 796 10.1038/nature01803 12917689

[B26] Brummelkamp TR Nijman SM Dirac AM Bernards R Loss of the cylindromatosis tumour suppressor inhibits apoptosis by activating NF-kappaB Nature 2003 424 797 801 10.1038/nature01811 12917690

[B27] Ninomiya-Tsuji J Kishimoto K Hiyama A Inoue J Cao Z Matsumoto K The kinase TAK1 can activate the NIK-I kappaB as well as the MAP kinase cascade in the IL-1 signalling pathway Nature 1999 398 252 256 10.1038/18465 10094049

[B28] Takaesu G Surabhi RM Park KJ Ninomiya-Tsuji J Matsumoto K Gaynor RB TAK1 is critical for IkappaB kinase-mediated activation of the NF-kappaB pathway J Mol Biol 2003 326 105 115 10.1016/S0022-2836(02)01404-3 12547194

[B29] Liu HH Xie M Schneider MD Chen ZJ Essential role of TAK1 in thymocyte development and activation Proc Natl Acad Sci USA 2006 103 11677 11682 10.1073/pnas.0603089103 16857737 PMC1544229

[B30] Sato S Sanjo H Takeda K Ninomiya-Tsuji J Yamamoto M Kawai T Matsumoto K Takeuchi O Akira S Essential function for the kinase TAK1 in innate and adaptive immune responses Nat Immunol 2005 6 1987 1095 10.1038/ni1255 16186825

[B31] Shim JH Xiao C Paschal AE Bailey ST Rao P Hayden MS Lee KY Bussey C Steckel M Tanaka N TAK1, but not TAB1 or TAB2, plays an essential role in multiple signaling pathways in vivo Genes Dev 2005 19 2668 2681 10.1101/gad.1360605 16260493 PMC1283960

[B32] Wan YY Chi H Xie M Schneider MD Flavell RA The kinase TAK1 integrates antigen and cytokine receptor signaling for T cell development, survival and function Nat Immunol 2006 7 851 858 16799562 10.1038/ni1355

[B33] Shambharkar PB Blonska M Pappu BP Li H You Y Sakurai H Darnay BG Hara H Penninger J Lin X Phosphorylation and ubiquitination of the IkappaB kinase complex by two distinct signaling pathways EMBO J 2007 26 1794 1805 10.1038/sj.emboj.7601622 17363905 PMC1847656

[B34] Wu X Sun SC Retroviral oncoprotein Tax deregulates NF-κB by activating Tak1 and mediating Tak1-IKK physical association EMBO Rep 2007 8 510 515 10.1038/sj.embor.7400931 17363973 PMC1866198

[B35] Sun SC Cesarman E NF-κB as a Target for Oncogenic Viruses Curr Top Microbiol Immunol 2011 349 197 244 10.1007/82_2010_108 PMC3855473 20845110

[B36] Yasunaga J Lin FC Lu X Jeang K-T Ubiquitin-specific peptidase 20 targets TRAF6 and human T cell leukemia virus type I Tax to negatively regulate NF-κB signaling J Virol 2011 85 6212 6219 10.1128/JVI.00079-11 21525354 PMC3126525

[B37] Xiao G Harhaj EW Sun S-C Domain-specific interaction with IKKγ is an essential step in Tax-mediated activation of IKK J Biol Chem 2000 275 34060 34067 10906125 10.1074/jbc.M002970200

[B38] Hutti JE Shen RR Abbott DW Zhou AY Sprott KM Asara JM Hahn WC Cantley LC Phosphorylation of the tumor suppressor CYLD by the breast cancer oncogene IKKepsilon promotes cell transformation Mol Cell 2009 34 461 472 10.1016/j.molcel.2009.04.031 19481526 PMC2746958

[B39] Grassmann R Berchtold S Radant I Alt M Fleckenstein B Sodroski JG Haseltine WA Ramstedt U Role of human T-cell leukemia virus type I X region proteins in immortalization of primary human lymphocytes in culture J Virol 1992 66 4570 4575 1351105 10.1128/jvi.66.7.4570-4575.1992 PMC241270

[B40] Smith MR Greene WC Identification of HTLV-1 tax transactivator mutants exhibiting novel transcriptional phenotypes Genes Dev 1990 4 1875 1885 10.1101/gad.4.11.1875 2276622

[B41] Xiao G Harhaj EW Sun SC NF-kappaB-inducing kinase regulates the processing of NF-kappaB2 p100 Mol Cell 2001 7 401 409 10.1016/S1097-2765(01)00187-3 11239468

[B42] Shibuya H Yamaguchi K Shirakabe K Tonegawa A Gotoh Y Ueno N Irie K Nishida E Matsumoto K TAB1: an activator of the TAK1 MAPKKK in TGF-beta signal transduction Sciece 1996 272 1179 1182 10.1126/science.272.5265.1179 8638164

[B43] Xiao G Cvijic ME Fong A Harhaj EW Uhlik MT Waterfield M Sun SC Retroviral oncoprotein Tax induces processing of NF-kappaB2/p100 in T cells: evidence for the involvement of IKKalpha EMBO J 2001 20 6805 6815 10.1093/emboj/20.23.6805 11726516 PMC125766

